# Prevalence of diphtheria and antimicrobial-resistant wound infections among asylum seekers in Heidelberg, Germany, August–October 2024

**DOI:** 10.1371/journal.pone.0350513

**Published:** 2026-06-09

**Authors:** Bernadette Walter, Katharina Lück, Maja Adam, Bettina Winter, Patryk Krauze, Jörg Rau, Nina Knab, Anne Kühn, Andreas Welker, Elisabeth Aichinger, Stefan Brockmann

**Affiliations:** 1 State Health Office, Ministry of Social Affairs, Health and Integration Baden-Wuerttemberg, Stuttgart, Germany; 2 Postgraduate Training for Applied Epidemiology (PAE), Department for Infectious Disease Epidemiology, Robert Koch Institute, Berlin, Germany; 3 ECDC Fellowship Programme, Field Epidemiology path (EPIET), European Centre for Disease Prevention and Control (ECDC), Stockholm, Sweden; 4 Local Health Authority Rhine-Neckar-District, Heidelberg, Germany; 5 Chemical and Veterinary Analysis Agency, Stuttgart, Germany; St Petersburg Pasteur Institute, RUSSIAN FEDERATION

## Abstract

**Purpose:**

In the second half of 2022 and 2023, an increase of diphtheria cases among asylum seekers was observed in Europe, with 120 cases reported in the state of Baden-Wuerttemberg, Germany. We aimed to determine prevalence of infection or colonization with toxigenic *Corynebacterium* among asylum seekers arriving between August and October 2024 in Heidelberg, Germany. In addition, we assessed antimicrobial-resistant wound infections and diphtheria immunity levels in this population.

**Methods:**

We conducted a cross-sectional study with a random sample of 1073 newly arrived asylum seekers. Consenting participants received a throat and, if applicable, wound swabs. Demographic, clinical and migration-related information was collected to identify potential risk groups. Throat swabs were tested for toxigenic *Corynebacterium* species, while wound swabs were additionally tested for other antimicrobial-resistant pathogens with relevance to therapeutic management. IgG antibody levels against diphtheria toxoid were quantified in serum samples of a random sub-sample.

**Results:**

Of the participants, 75% were male (n = 804), and ages ranged from 15 to 60 years (median = 27 years). The most common nationalities were Syrian (n = 281), Turkish (n = 189) and Afghan (n = 117). No toxigenic *Corynebacterium* was identified. Thirty-seven participants carried inflamed wounds, of whom 18 (49%) had wounds infected with methicillin-resistant *Staphylococcus aureus* (MRSA), corresponding to 1.7% (95% CI 1.0–2.6%) of the entire study population. Overall, 38.0% (95% CI 33.3–44.8%) showed non-protective diphtheria antibody titers, while acceptance of the on-site diphtheria vaccination was high at 93% (n = 998).

**Conclusion:**

While no toxigenic *Corynebacterium* was detected, low immunity levels and antimicrobial resistance findings underline the importance of early diagnosis, treatment and vaccination options in displaced populations.

## Introduction

Diphtheria is a vaccine-preventable infectious disease caused by toxigenic strains of *Corynebacterium* species. In recent years before 2022, diphtheria had become a rare disease in both Europe and Germany. Between 2011 and 2021, annual reported case numbers ranged from 20 to 66 across Europe and from 4 to 26 in Germany [1–3]. In Germany, cases occurred predominantly sporadically and were mainly associated with cutaneous infections caused by *C. ulcerans* [4]. Reports of local transmission were limited to isolated occasions [5]. In 2022–2023, diphtheria reemerged in several European countries [6]. Germany reported 310 cases, including 126 in the federal state of Baden-Wuerttemberg. The majority of cases were caused by *C. diphtheriae* and occurred among asylum seekers [7]. Most cases presented with cutaneous infections, though some individuals suffered from respiratory or both manifestations [8]. In case of cutaneous diphtheria, co-infections with *Staphylococcus aureus*, including strains with methicillin resistance (MRSA), and *Streptococcus pyogenes* were reported [9]. Previously, wound infections with extended-spectrum β-lactamase (ESBL)-producing Enterobacterales have also been reported among asylum seekers [10]. In addition, higher carriage rates of MRSA and other antimicrobial-resistant bacteria have been described among asylum seeker and refugee patients in comparison to the general patient population [10,11]. Migration history was found to be associated with presence of Panton–Valentine leukocidin (PVL), which is a virulence factor produced by certain strains of *S. aureus* that is associated with recurrent skin and soft tissue infections as well as severe necrotizing disease [12,13]. PVL formation is independent of the methicillin resistance of *S. aureus*.

At the same time, vaccination records among asylum seekers are frequently unavailable and thus information on immunity against diphtheria in this population is rare [9]. If vaccination status is uncertain, the German Standing Committee on Vaccination (STIKO) recommends catch-up immunization against diphtheria at all ages for both the general population and asylum seekers, alongside other age- and risk-indicated vaccinations [14].

Recent observations suggested diphtheria transmission beyond the asylum-seeking population in Germany. In 2023, several cases of diphtheria occurred also among persons experiencing homelessness in Frankfurt, Germany. Identification of sequence type 574 (ST-574) in case-isolates suggested a possible epidemiological link to an earlier diphtheria cluster detected among asylum seekers in 2022 [15]. A second sub-cluster of ST-574 was identified in Germany since 2024, associated with cases of cutaneous and respiratory diphtheria, including three fatalities [16].

Besides symptomatic cases, studies have also reported asymptomatic respiratory carriage of *C. diphtheriae* among asylum seekers [17,18]. There is evidence that asymptomatic respiratory carriage can lead to disease transmission [19]. This highlights the potential for silent transmission. However, previous investigations have focused on symptomatic individuals or asymptomatic close contacts, which may result in an underestimation of absolute case numbers and risk.

To address this, we conducted a cross-sectional study between August – October 2024 in a cohort of newly arrived asylum seekers in Heidelberg, Germany. Throat swabs were analyzed for toxigenic *Corynebacteria*. Wounds were screened for toxigenic *Corynebacteria* and antibiotic-resistant bacteria targeted by our screening approach, with a focus on pathogens associated with limited treatment options due to antimicrobial resistance. A random sub-sample of participants was serologically tested for IgG antibodies against diphtheria toxoid. This study aimed to provide evidence on the prevalence of diphtheria, its immunity levels and antibiotic-resistant wound infections in newly arrived asylum seekers. The findings could inform targeted vaccination and infection control strategies in reception facilities and support the clinical management in routine health care.

## Methods

### Study design

The study was conducted at a reception center in Heidelberg, Germany between August 12^th^ and October 31^st^ 2024. Individuals with a minimum age of 15 years were eligible for inclusion. During the study period, 2066 asylum seekers met this criterion. Simple random sampling was used to recruit potential study participants. Of 1250 eligible individuals approached, 177 declined participations, while 1073 provided written informed consent after receiving appropriate information on the study (S1 Fig). In addition, for persons aged under 18, their legal representative had to agree to the consent declarations of the minor in written. This study received ethical approval from the State Medical Chamber of Baden-Wuerttemberg (F-2024–046).

### Collected data and samples

During the study examination demographic data (age, sex, nationality) was collected. The throat was examined for inflammation and a throat swab was taken of every participant. Head and limbs were examined for wounds, which were swabbed. Inflammation of wounds was assessed based on clinical signs such as redness, swelling, discharge or pain. The subsequent detection of pathogens in wounds clinically assessed as inflamed was classified as infection. Information was collected whether the participant received a diphtheria vaccination offered on site. All study participants were also eligible for serological investigations. Blood samples were taken from a simple random sub-sample of 263 participants. 27 serum samples resulted from a piloting week of the study between July 22^nd^ and 26^th^ 2024.

### Laboratory swab investigations

The throat and wound swabs were spread on tellurite and blood agar plates (with 50 µg fosfomycin disks). Colonies suspected of being *Corynebacterium* were examined for catalase activity, Gram-stained and identified by matrix-assisted laser desorption/ionization time-of-flight mass spectrometry (MALDI-TOF MS, Bruker Corporation, Billerica, USA) with an extended database [20]. Potential toxin-producing *Corynebacterium* isolates (*C. diphtheria* and *C. ulcerans*) were forwarded to the consultant laboratory for diphtheria at the Bavarian Health and Food Safety Authority (LGL) in Oberschleißheim, Germany, where toxin production testing, toxin gene detection and antimicrobial resistance testing were performed according to the WHO laboratory manual for the diagnosis of diphtheria and other related infections [21].

Wound swabs were in addition spread on MacConkey agar plates and selective agar plates for drug resistant pathogens, including MRSA (CHROMID MRSA agar bioMérieux, Marcy-l’Étoile, France), vancomycin-resistant enterococci (VRE) (CHROMID VRE agar bioMérieux, Marcy-l’Étoile, France), extended-spectrum β-lactamase (ESBL)- or carbapenemase-producing organisms (CHROMID CARBA smart agar bioMérieux, Marcy-l’Étoile, France). These media can detect resistant pathogens identified in skin and soft tissue infections, including MRSA, *Enterococcus faecium* and *Enterococcus faecalis* (VRE), as well as multidrug-resistant Gram-negative organisms such as *Escherichia coli*, *Enterobacter* spp. (e.g., *Enterobacter cloacae*) and other *Enterobacterales* (e.g., *Proteus mirabilis*). Suspect colonies were biochemically identified using VITEK 2 Compact (bioMérieux) and an antimicrobial susceptibility testing (AST) was performed and assessed according to EUCAST 2024 guidelines. Colonies suspected to be MRSA were further analyzed by polymerase chain reaction (PCR) for the *mecA/mecC* gene (diarellaMRSA 3.0 TM, gerbion, Kornwestheim, Germany). PVL genes (*lukS* and *lukF*) were detected using a commercial assay according to the manufacturer’s protocol (GenoType MRSA, Hain Lifescience, Nehren, Germany).

### Sequencing of MRSA isolates

Genomic DNA of *S. aureus* isolates was isolated as described [22]. Next, the sequencing library was prepared using the NEBNext® Ultra™ II FS DNA Library Prep Kit for Illumina, following the manufacturer’s instructions. Subsequently, the isolates were sequenced on a MiSeq system in a paired-end mode with a read length of 2 times 250 bp. For the processing of raw genomic data and further analysis, MBioSEQ Ridom Typer (Ridom GmbH, Germany) was used. Following quality control with FASTQC and processing with Trimmomatic [23] to remove Illumina adapters and quality trim the reads, the genomes were assembled using SKESA 2.3.0 [24]. Assemblies were analyzed and compared using core genome multilocus sequence typing (cgMLST) [25]. AMRFinder Plus (version 4.0.15) [26] was used to screen the genomes for genes encoding antimicrobial resistances. Sequencing data of MRSA isolates were submitted to the European Nucleotide Archive (http://www.ebi.ac.uk/ena) under the accession number PRJEB98196.

### Quantification of IgG antibodies against diphtheria toxoid

Levels of IgG antibodies against diphtheria toxoid were measured using an enzyme-linked immunosorbent assay (ELISA) (SERION ELISA classic Diphtheria IgG), following the manufacturer’s instructions. Results were categorized according to the World Health Organization as follows [27]:

<0.1 IU/ml: insufficient immunity

0.1 - ≤ 1 IU/ml: sufficient immunity

>1 IU/ml: long-term immunity

### Statistical analysis

Data were analyzed using R software (version 4.4.0). Confidence intervals (CI) for prevalence represent exact Clopper-Pearson intervals. Observations with “unknown” or “other” category for sex were excluded prior to regression analysis. Age was included as a continuous variable in regression models to preserve statistical power and avoid sparse data issues. A p-value <0.05 was considered to be statistically significant. To assess the association between independent variables and the outcome of MRSA infected wounds, a multivariable analysis was performed. To account for the low number of MRSA cases and potential separation, penalized logistic regression using Firth logistic regression was performed [28]. Analyses were conducted using the logistf package in R. Age and sex were included as independent variables in the multivariable model. For serological analyses, antibody titers were dichotomized by combining values ≥0.1 IU/ml into a single category. The association between age and sex with low antibody titers <0.1 IU/ml was assessed using univariable logistic regression models fitted with the glm function from the R stats package. Geometric mean concentrations (GMCs) of antibody levels were calculated by log-transforming the data, calculating means and 95% CIs on the log scale, and back-transforming to the original scale.

## Results

### Participant characteristics and microbiological findings

**Fig 1:**
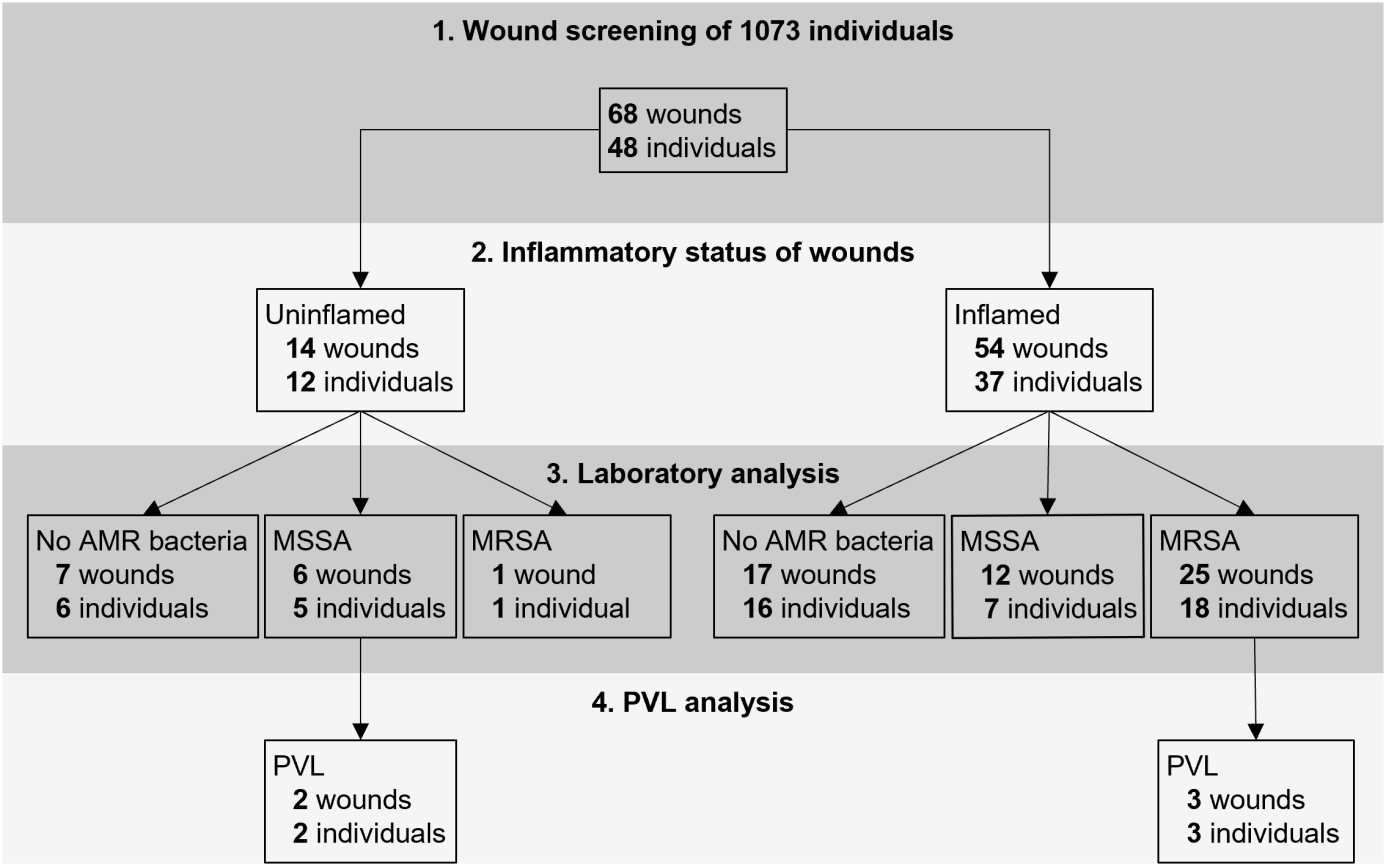
Wound screening among asylum seekers in Heidelberg, Germany, August–October 2024. In this study asylum seekers arriving in Heidelberg, Germany, August – October 2024 were systematically screened for wounds. Inflammatory status of wounds was assessed based on clinical signs of inflammation. Wound swabs were tested for antimicrobial-resistant pathogens. The presence of the Panton-Valentine leukocidin (PVL) gene was confirmed in methicillin-resistant and methicillin-susceptible *Staphylococcus aureus* samples. Some participants had multiple wounds and may appear in more than one category. One participant had both an inflamed and an uninflamed wound, with no antimicrobial-resistant bacteria identified in either wound. Furthermore, four participants presenting inflamed wounds had at least two wounds that were assigned to different diagnostic categories following laboratory analysis. AMR: Antimicrobial resistance. MSSA: Methicillin-susceptible *Staphylococcus aureus*. MRSA: Methicillin-resistant *Staphylococcus aureus*.

The median age of the participants was 27 years (IQR: 22–36). The majority of participants were male (75%). Most common nationalities were Syrian, Turkish and Afghan (Table 1). In total, 68 wounds were identified on 48 participants. Among these, 54 wounds from 37 individuals were assessed as inflamed (Fig 1). This corresponds to an inflamed wound prevalence of 3.4% [95% CI: 2.4–4.7%]. The detection of pathogens in wounds clinically assessed as inflamed was classified as infection.

**Table 1:** Prevalence of MRSA-wound infections stratified by age, sex and nationality among asylum seekers arriving in Heidelberg, Germany, August – October 2024.

	Total		Participants with MRSA wound infection		
	No.	%	No.	%	[95% CI]
Age groups (y)					
15–20	205	19.1	4	2.0	[0.5–4.9]
21–30	452	42.1	12	2.7	[1.4–4.6]
31–40	245	22.8	2	0.8	[0.1–2.9]
41–50	121	11.2	0	0	[0–3]
51–60	50	4.7	0	0	[0–7.1]
>60	0	0	0	0	
Sex					
M	804	74.9	18	2.2	[1.3–3.5]
F	266	24.8	0	0	[0–1.4]
Other / Unknown	3	0.3		0	[0–70.8]
Nationality					
Syrian	281	26.2	10	3.6	[1.7–6.4]
Turkish	189	17.6	≤3^b^	≤1.6	
Afghan	117	10.9	≤3^b^	≤2.6	
Macedonian	54	5.0	0	0	[0–6.6]
Algerian	39	3.6	≤3^b^	≤7.7	
Chinese	38	3.5	0	0	[0–9.3]
Kosovar	34	3.2	0	0	[0–10.3]
Colombian	32	3.0	0	0	[0–10.9]
Tunisian	25	2.3	0	0	[0–13.7]
Serbian	23	2.1	0	0	[0–14.8]
Iranian	22	2.1	0	0	[0–15.4]
Nigerian	21	2.0	0	0	[0–16.1]
Georgian	20	1.9	0	0	[0–16.8]
Cameroonian	16	1.5	0	0	[0–20.6]
Moroccan	16	1.5	0	0	[0–20.6]
Indian	14	1.3	0	0	[0–23.2]
Somali	14	1.3	0	0	[0–23.2]
Iraqi	13	1.2	≤3^b^	≤23.1	
Russian	13	1.2	0	0	[0–24.7]
Guinean	11	1.0	0	0	[0–28.5]
Sri Lankan	10	0.9	0	0	[0–30.8]
Other / Unkown	71	6.6	0	0	[0–5.1]
Total	1073	100	18	1.7	[1.0–2.6]

^a^Nationalities with No. < 10 shown as “Other”.

^b^Nationalities with ≤3 participants with MRSA wound infection are reported with reduced precision for anonymization purposes.

One throat swab of an asymptomatic participant was identified with *C. diphtheriae*. The isolate was toxin gene negative during subsequent PCR. No other non-toxigenic or toxigenic *Corynebacterium spp*. was detected in any throat or wound swab. MRSA was detected in 25 inflamed wounds from 18 individuals, representing approximately half of all inflamed wounds. This corresponds to a prevalence of MRSA wound infections of 1.7% [95% CI: 1.0–2.6%] among the entire study population (Table 1). PVL was identified in MRSA infected wounds of three individuals. In multivariable analysis including sex and age as independent variables, female sex was associated with lower odds of a MRSA wound infection (odds ratio (OR) = 0.09, 95% CI 0.001–0.70, p = 0.014), while increasing age was associated with decreased odds (OR = 0.93 per year, 95% CI 0.86–0.99, p = 0.014). The overall model was statistically significant (likelihood ratio test p = 0.001).

Methicillin-susceptible Staphylococcus aureus (MSSA) was detected in 12 inflamed wounds from 7 individuals. Additionally, 14 wounds from 12 individuals were assessed to be not inflamed. MRSA was detected in one of these wounds. MSSA was found in six non-inflamed wounds from five individuals, and in two of these, the PVL gene was present. These results, without clinical signs of inflammation, may reflect colonization rather than infection. No VRE or ESBL or carbapenemase-producing pathogens were identified in wound swabs.

### Antimicrobial resistances among MRSA isolates

Antimicrobial susceptibility testing of MRSA isolates from 14 participants with inflamed wounds revealed high resistance rates to fusidic acid, clindamycin and tetracycline (Table 2). Two isolates showed phenotypic resistance to trimethoprim/sulfamethoxazole.

**Table 2:** Number and frequency of specific antibiotic resistances detected in MRSA isolates (n = 14) from inflamed skin wounds of asylum seekers arriving in Heidelberg, Germany, August – October 2024 based on phenotypic antimicrobial susceptibility testing.

Antibiotic	Interpretation	No.	%
Cefoxitin Screen			
	Positive	14	100
Benzylpenicillin			
	Resistant	14	100
Oxacillin			
	Resistant	14	100
Gentamicin			
	Resistant	4	29
	Susceptible	10	71
Tobramycin			
	Resistant	3	21
	Susceptible	11	79
Levofloxacin			
	Resistant	2	14
	Intermediate	12	86
Inducible Clindamycin resistance			
	Positive	5	36
	Negative	9	64
Clindamycin			
	Resistant	5	36
	Susceptible	9	64
Linezolid			
	Susceptible	14	100
Teicoplanin			
	Susceptible	14	100
Vancomycin			
	Susceptible	14	100
Tetracycline			
	Resistant	5	36
	Susceptible	9	64
Tigecycline			
	Susceptible	14	100
Fosfomycin			
	Resistant	1	7
	Susceptible	13	93
Fusidic Acid			
	Resistant	10	71
	Susceptible	4	29
Trimethoprim/Sulfamethoxazole			
	Resistant	2	14
	Susceptible	12	86

Phylogenetic analysis using core genome multi locus sequence typing (cgMLST) demonstrated high genetic diversity, with pairwise allelic distances across the minimum spanning tree ranging from 88 to 1,655 (S2 Fig). All isolates harbored resistance genes against erythromycin, chloramphenicol, tetracycline and tigecycline (S3 Table).

### Diphtheria immunity levels

Immunity levels against diphtheria were analyzed from serum samples of 290 participants (Table 3). Overall, 7% of individuals showed high antibody concentrations (>1.0 IU/mL), indicating long-term immunity. The majority of participants (54%) had antibody levels in the range of 0.1–1.0 IU/mL, which are considered protective. A total of 38% had low antibody levels (<0.1 IU/mL), indicating incomplete protection against diphtheria. Univariable logistic regression analysis showed increasing odds of low antibody levels (<0.1 IU/mL) with increasing age (OR 1.03 per year, 95% CI 1.01–1.06, p = 0.011). There was no evidence of an association between sex and low antibody levels (<0.1 IU/mL) (OR 1.03, 95% CI 0.59–1.80; p = 0.91). Due to small numbers in several nationality categories, nationality was not included in the regression analysis to avoid sparse data bias.

**Table 3:** IgG antibody levels against diphtheria toxoid among asylum seekers arriving in Heidelberg, Germany, August – October 2024 stratified by age groups, sex, and nationality.

	Total		<0.1 IU/ml			0.1– ≤ 1.0 IU/ml			>1.0 IU/ml		
				Prevalence			Prevalence			Prevalence	
	No.	%	No.	%	[95% CI]	No.	%	[95% CI]	No.	%	[95% CI]
Age groups (y)											
15–20	56	19.3	19	33.9	[21.8–47.9]	32	57.1	[43.2–70.3]	5	8.9	[2.9–19.6]
21–30	123	42.4	41	33.3	[25.1–42.4]	73	59.3	[50.1–68.1]	9	7.3	[3.4–13.4]
31–40	65	22.4	27	41.5	[29.4–54.4]	32	49.2	[36.6–61.9]	6	9.2	[3.5–19.0]
41–50	34	11.7	17	50.0	[32.4–67.6]	17	50.0	[32.4–67.6]	0	0	[0.0–10.3]
51–60	12	4.1	9	75.0	[42.8–94.5]	2	16.7	[2.1–48.4]	1	8.3	[0.2–38.5]
>60	0	0									
Sex											
M	222	76.6	86	38.7	[32.2–45.5]	119	53.6	[46.8–60.3]	17	7.7	[4.5–12.0]
F	68	23.4	27	39.7	[28.0–52.3]	37	54.4	[41.9–66.5]	4	5.8	[1.6–14.4]
Nationality											
Syrian	82	28.2	37	45.1	[34.1–56.5]	44	53.7	[42.2–64.7]	1	1.2	[0.0–6.6]
Turkish	43	14.8	9	20.9	[10.0–36.0]	29	67.4	[51.5–80.9]	5	11.6	[3.9–25.1]
Afghan	30	10.3	19	63.3	[43.9–80.1]	9	30.0	[14.7–49.4]	2	6.7	[0.8–22.1]
Macedonian	19	6.6	2	10.5	[1.3–33.1]	15	78.9	[54.4–93.9]	2	10.5	[1.3–33.1]
Kosovar	12	4.1	5	41.7	[15.2–72.3]	7	58.3	[27.7–84.8]	0	0.0	[0.0–26.5]
Chinese	10	3.4	6	60.0	[26.2–87.8]	3	30.0	[6.7–65.2]	1	10.0	[0.3–44.5]
Other^a^	94	32.4									
Total	290	100	113	38.0	[33.3–44.8]	156	53.8	[47.9–59.6]	21	7.2	[4.5–10.9]

^a^Nationalities with <10 serum samples.

The overall Geometric Mean Concentration (GMC) was 0.153 IU/mL (S4 Table). Of 1073 study participants, 996 (~93%) received a vaccination against diphtheria during the health examination in the reception center.

## Discussion

A recent diphtheria outbreak among asylum seekers in Europe peaked in 2022–2023, with most confirmed cases reported by Germany [7]. Cases were mainly cutaneous and limited to reception centers. By 2024–2025, reported diphtheria cases in asylum seeker populations had declined sharply [29]. This is consistent with our findings: no symptomatic infection or asymptomatic colonization with toxigenic *Corynebacteria* was detected during our study. We identified one asymptomatic participant with non-toxigenic *C. diphtheriae* in a throat swab. Non-toxigenic *C. diphtheriae* has been previously isolated among asylum seekers in UK, Switzerland and Germany [17,30]. Infections caused by non-toxigenic strains do not result in classical toxin-mediated diphtheria. However, they can still lead to severe invasive disease, particularly among vulnerable populations [31]. A cross-sectional study conducted in Denmark 2016–2018, prior to the emergence of diphtheria among asylum seekers in Europe, did also not identify *C. diphtheriae* among 104 throat swaps of asymptomatic Syrian refugees [32]. Diphtheria prevalence among asylum seekers arriving in England 2022 was estimated to be 0.15% [17]. However, this estimate was derived from surveillance data, which primarily capture symptomatic disease and may therefore underestimate the true prevalence of infection and colonization during the corresponding period. The decline in cases by 2024 may relate to reduced migration: Frontex reported a 78% drop in irregular border crossings along the Western Balkan route and a 38% overall decline in illegal entries to the EU from 2023 to 2024 [33]. This substantial reduction of migration, particularly through Balkan countries, may have contributed to fewer transmission opportunities and a subsequent decline in diphtheria incidence. In addition, increased awareness following the rise in cases in 2022 and 2023, together with the potential implementation of recommendations issued by the European Centre for Disease Prevention and Control (ECDC), may have contributed to the prevention as well as earlier diagnosis and treatment of infections [7]. Notably, our serological results indicate persisting immunity gaps in the studied population, particularly among nationalities most affected in 2022 and 2023, suggesting that vaccination coverage remained incomplete. In contrast, autochthonous diphtheria cases have emerged in Germany since mid-2023 [16]. Afghanistan, one of the main countries of origin, reported 207 diphtheria cases in 2024, indicating a remaining risk for imported cases [34].

Serological testing showed that only 7% of participants had long-term immunity, while 39% showed non-protective titers. The overall GMC was 0.153 IU/mL. Antibody titers above 0.1 IU/mL are considered protective. However, it should be noted that ELISA assays primarily quantify binding antibodies and may not fully reflect functional neutralizing capacity. Previous studies have shown that ELISA measurements overestimate diphtheria antibody titers particularly below 0.1 IU/mL in comparison to *in vitro* toxin neutralization tests. Consequently, the actual GMC may be lower than suggested [21]. Immunity declined with age, which mirrors trends in displaced but also in European populations [35,36]. However, 93% of participants accepted diphtheria vaccination during their health screening, underlining the potential of low-threshold vaccination offers.

Although no diphtheria case was detected, 1.7% of participants had MRSA-infected wounds, all being male. Other studies have highlighted increased rates of MRSA carriage and infection among migrants in Europe in comparison to the general population. The MRSA prevalence among forced migrants was estimated to be 11% [37]. At the same time, evidence from Germany suggests that although colonization with resistant organisms may be common among refugees, clinically manifest resistant skin infections remain relatively uncommon: in a pediatric refugee cohort, 22 of 325 hospitalized children were colonized with MRSA, but only three had MRSA-associated skin and soft tissue infections [38]. Literature on antimicrobial-resistant skin and tissue infections among asylum seekers in Europe remains scarce. In a Finnish study between 2010 and 2017, resistant wound infections were predominantly caused by MRSA: among 447 asylum seekers screened during hospital admission, 97 (21.3%) were colonized with MRSA and four presented with MRSA wound infections, while only two ESBL-associated wound infections caused by *Enterobacter cloacae* and *Proteus mirabilis* were identified. [10]. These findings are consistent with our results, in which MRSA represented the only resistant pathogen detected in wounds. In addition to *C. diphtheriae* and *S. aureus*, *S. pyogenes* has been reported in wound infections among asylum seekers [9,39]. However, because our microbiological screening was restricted to organisms with antimicrobial resistances relevant to therapeutic management, we are unable to draw conclusions regarding the prevalence of susceptible organisms such as *S. pyogenes* in this cohort. In our study, 5 of 44 *S.aureus* isolates (11%) were positive for PVL, which is markedly lower than the 40–51.1% reported in two German ambulatory care studies of skin and soft tissue infections [13,40]. This considerable difference might partly be related to the different study settings as our data is derived from wound screening rather than clinically presented infections. A Dutch study among asylum seekers found PVL in 42% of MRSA isolates [11]. However, these isolates were obtained not only from wound swabs but also from broader screening samples such as nasal swabs limiting direct comparability.

According to the cgMLST scheme of *S. aureus,* a maximum allelic distance of 24 alleles is used to define a complex type [41]. The supporting study shows a highly probable common origin of different *S. aureus* isolates (up to 18 allelic differences) in a nosocomial setting [25], taking into account the mutation rate for MRSA of one nucleotide per six weeks [42]. Therefore, for a close genetic relationship, we would expect < 24 allelic differences among the isolates. However, the phylogenetic analysis revealed no close genetic relationship among the isolates, with the closest allelic distance being 88. This indicates probably independent origins. Isolates displayed multiple resistances against antibiotics and resistance rates were considerably higher than those reported among inpatients in Germany [43].

Our findings underscore the importance of timely diagnostic and treatment options for arriving asylum seekers, considering potential antibiotic resistances. Proactive vaccination offers can be an effective measure to close immunity gaps.

## Supporting information

S1 Fig Flowchart of participant recruitment and sample collection procedure. (PDF)

S2 Fig Phylogenetic analysis of the 14 sequenced Staphylococcus aureus isolates. For cluster analysis, a minimum spanning tree was generated based on core genome multilocus sequence typing (cgMLST) comparing a total of 1,861 genes. The numbers on top of the circles represent the internal laboratory sample numbers. The number on each connecting line indicates the number of allelic differences between the respective isolates.
(PDF)

S3 Table Number and frequency of specific antibiotic resistances detected in MRSA isolates (n = 14) from skin wounds of asylum seekers arriving in Heidelberg, Germany, August – October 2024 based on resistance gene detection. (PDF)

S4 Table Geometric mean concentrations (GMC) of anti-diphtheria toxoid antibodies stratified by age, sex and nationality. (PDF)
