# Supplementary figures and images for: Prevalence of diphtheria and antimicrobial-resistant wound infections among asylum seekers in Heidelberg, Germany, August–October 2024
Source: PLoS One. 2026 Jun 9;21(6):e0350513. doi: 10.1371/journal.pone.0350513 (PMC13249197; doi:10.1371/journal.pone.0350513)

**S1 Fig. Flowchart of participant recruitment and sample collection procedure.**

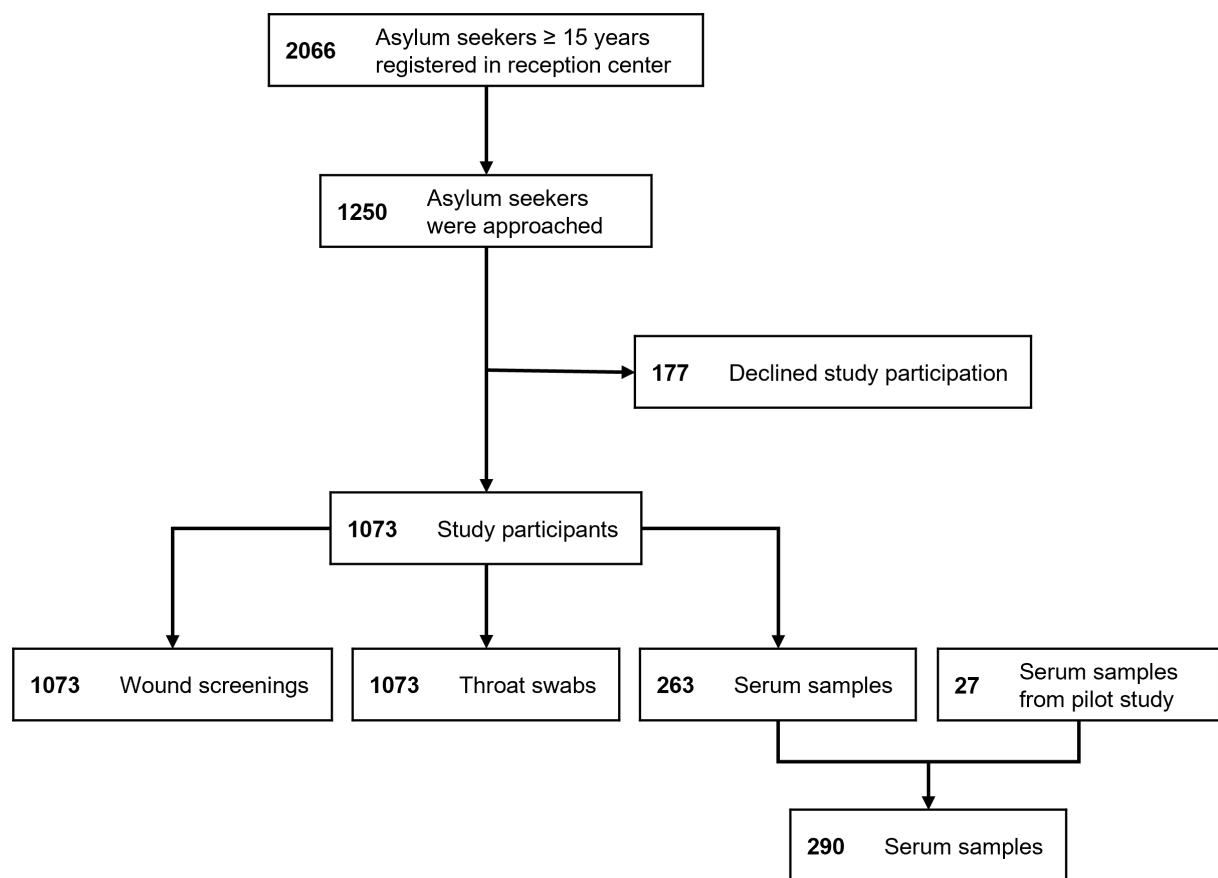

Supplement: S1 Fig — (PDF) [file pone.0350513.s001.pdf]
