# Supplementary material for: Prevalence of diphtheria and antimicrobial-resistant wound infections among asylum seekers in Heidelberg, Germany, August–October 2024
Source: PLoS One. 2026 Jun 9;21(6):e0350513. doi: 10.1371/journal.pone.0350513 (PMC13249197; doi:10.1371/journal.pone.0350513)

S2 Fig. Phylogenetic analysis of the 14 sequenced *Staphylococcus aureus* isolates. For cluster analysis, a minimum spanning tree was generated based on core genome multilocus sequence typing (cgMLST) comparing a total of 1,861 genes. The numbers on top of the circles represent the internal laboratory sample numbers. The number on each connecting line indicates the number of allelic differences between the respective isolates.

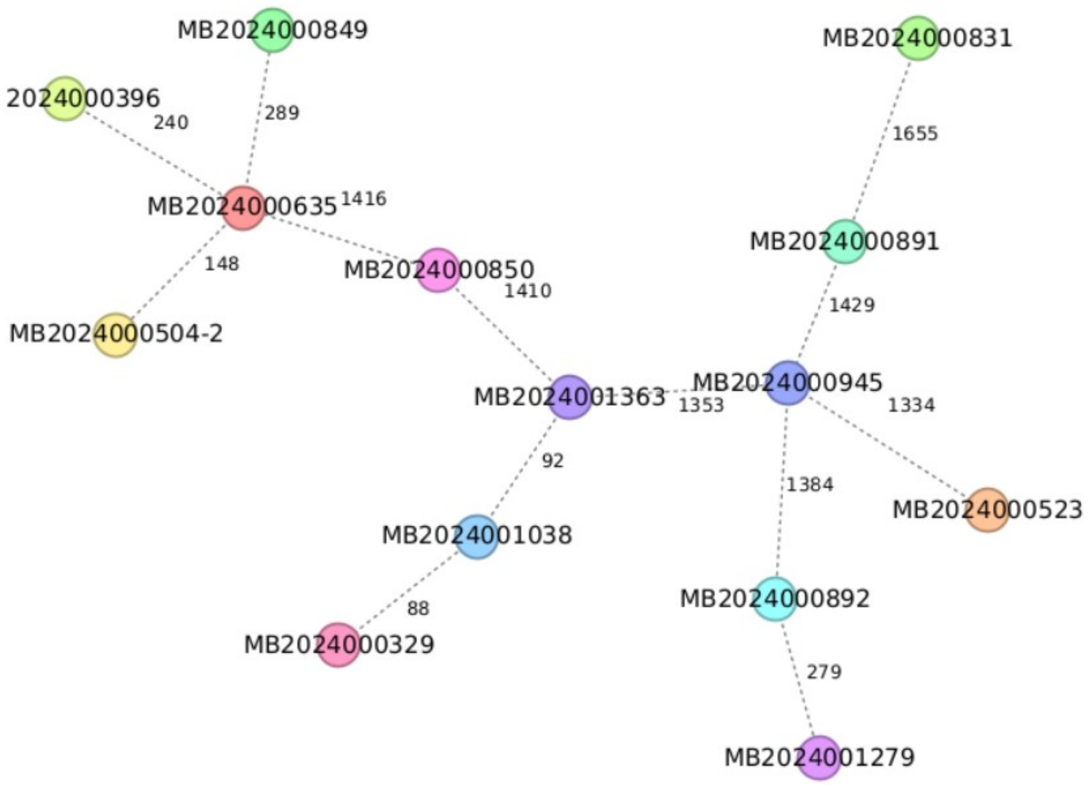

Supplement: S2 Fig — For cluster analysis, a minimum spanning tree was generated based on core genome multilocus sequence typing (cgMLST) comparing a total of 1,861 genes. The numbers on top of the circles represent the internal laboratory sample numbers. The number on each connecting line indicates the number of allelic differences between the respective isolates. (PDF) [file pone.0350513.s002.pdf]
