# Supplementary material for: Prevalence of diphtheria and antimicrobial-resistant wound infections among asylum seekers in Heidelberg, Germany, August–October 2024
Source: PLoS One. 2026 Jun 9;21(6):e0350513. doi: 10.1371/journal.pone.0350513 (PMC13249197; doi:10.1371/journal.pone.0350513)
Supplement: S3 Table — (PDF) [file pone.0350513.s003.pdf]

S3 Table. Number and frequency of specific antibiotic resistances detected in MRSA isolates (n = 14) from skin wounds of asylum seekers arriving in Heidelberg, Germany, August – October 2024 based on resistance gene detection.

Antibiotic	Determinant of resistance	No.	%
**Methicillin**		**14**	**100**
	mecA	14	100
**Erythromycin**		**14**	**100**
	lmrS	14	100
	erm(C)	4	29
	erm(B)	1	7
**Chloramphenicol**		**14**	**100**
	lmrS	14	10
	fexA	2	14
**Tetracycline**		**14**	**100**
	tet(38)	14	100
	tet(M)	2	14
	tet(K)	2	14
	tet(L)	1	7
**Tigecycline**		**14**	**100**
	mepA	14	100
**Beta-lactam**		**12**	**86**
	blaI	12	86
	blaR1	10	71
	blaZ	6	43
	blaPC1	2	14
**Fusidic Acid**		**10**	**71**
	fusC	8	57
	fusA_L461K	1	7
	fusA_V90I	1	7
**Fosfomycin**		**7**	**50**
	fosB	5	36
	fosY	1	7
	glpT_A100V	1	7
	murA_E291D / T396N	1	7
**Amikacin / Kanamycin**		**5**	**36**
	aac(6')-Ie/aph(2'')-Ia	4	29
	aph(3')-IIIa	4	29
**Clindamycin / Streptogramin B**		**5**	**36**
	erm(C)	4	29
	erm(B)	1	7
**Gentamicin / Tobramycin**		**4**	**29**
	aac(6')-Ie/aph(2'')-Ia	4	29
**Streptomycin**		**4**	**29**
	ant(6)-Ia	2	14
	str	1	7
**Tylosin**		**4**	**29**
	erm(C)	4	29
**Quinolone**		**4**	**29**
	parC_S80F	4	29
	gyrA_S84L	2	14
**Streptothricin**		**4**	**29**
	sat4	4	29
**Trimethoprim**		**3**	**21**
	dfrB_F99Y	1	7
	dfrE	1	7
	dfrG	1	7
	dfrS1	1	7
**Florfenicol**		**2**	**14**
	fexA	2	14
**Cephalosporin**		**1**	**7**
	pbp4_R200L	1	7
