# Supplementary material for: Prevalence of diphtheria and antimicrobial-resistant wound infections among asylum seekers in Heidelberg, Germany, August–October 2024
Source: PLoS One. 2026 Jun 9;21(6):e0350513. doi: 10.1371/journal.pone.0350513 (PMC13249197; doi:10.1371/journal.pone.0350513)
Supplement: S4 Table — (PDF) [file pone.0350513.s004.pdf]

S4 Table. Geometric mean concentrations (GMC) of anti-diphtheria toxoid antibodies stratified by age, sex and nationality.

N = 290

		No.	%	GMC in IU/mL	[95% CI]
Age groups (y)					
	15–20	56	19.3	0.162	[0.113–0.234]
	21–30	123	42.4	0.176	[0.140–0.222]
	31–40	65	22.4	0.153	[0.117–0.200]
	41–50	34	11.7	0.106	[0.077–0.146]
	51–60	12	4.1	0.072	[0.033–0.158]
	>60	0	0		
Sex					
	M	222	76.6	0.160	[0.136–0.189]
	F	68	23.4	0.130	[0.098–0.172]
Nationality					
	Syrian	82	28.2	0.119	[0.094–0.149]
	Turkish	43	14.8	0.253	[0.178–0.360]
	Afghan	30	10.3	0.081	[0.049–0.133]
	Macedonian	19	6.6	0.280	[0.178–0.442]
	Kosovar	12	4.1	0.107	[0.045–0.256]
	Chinese	10	3.4	0.088	[0.038–0.206]
	Other*	94	32.4		
Total		290	100	0.153	[0.132–0.176]

*Nationalities with <10 serum samples.
